# Changes and determinants of pneumococcal vaccine uptake in Ethiopia

**DOI:** 10.1371/journal.pgph.0004192

**Published:** 2025-01-22

**Authors:** Biniyam Tedla Mamo, Ferehiwot Gebrehiwot Geram, Kebron Yihenew Getnet, Zelalem Tazu Bonger

**Affiliations:** 1 Haramaya University College of Health and Medical Science, Harar, Ethiopia; 2 Ohio State Global One Health Initiative, LLC, Addis Ababa, Ethiopia; 3 Washington Medical Center, Addis Ababa, Ethiopia; 4 Karamara General Hospital, Jigjig, Ethiopia; Murdoch Children's Research Institute, AUSTRALIA

## Abstract

Pneumococcal pneumonia is one of the most common causes of severe pneumonia and pneumonia-related mortality globally. It ranked among the leading causes of morbidity and mortality in children under five years in Ethiopia. Vaccination reduces the burden of pneumonia and pneumococcal infections in both children and adults. This study assesses changes in pneumococcal vaccine coverage over time and identifies factors associated with the vaccine uptake. The study was based on secondary data from the Ethiopian Demographic and Health Surveys (EDHS) in 2016 and 2019, involving 1,929 children in 2016 and 1,008 in 2019, aged 12–23 months. A cross-sectional study design was conducted. The percentage change in pneumococcal conjugate vaccine (PCV) coverage was used to quantify the degree of change over time, while multilevel ordinal logistic regression identifies significant factors. All statistical tests were performed using a 5% significance threshold. The study found a significant 21.8% (95% CI: 9.8-35.2) change in the proportion of children receiving complete doses of PCV, from 49.1% in 2016 to 59.8% in 2019. Children in rural areas were 69% less likely to receive more doses of PCV vaccinations than those living in urban areas (AOR = 0.307, 95% CI: 0.127 - 0.742). Second or higher-order births were associated with greater uptake doses of PCV (AOR = 2.519, 95% CI: 1.143-5.548). Child born in health facilities were 2.35 times more likely to receive full vaccination than those born at home (AOR = 2.350, 95% CI: 1.132-4.882). Additionally, children whose mothers had more antenatal care (ANC) visits were more likely to complete their pneumococcal vaccination. Despite the increase in uptake, Ethiopia remains far from reaching its immunization goals. The study showed that place of residence, birth order, place of delivery, antenatal care and regional variation were significantly associated with pneumococcal vaccine uptake.

## Introduction

The development and introduction of new vaccinations and the expansion of immunization programs to reach every child has made significant strides in the last 10 years. This advancement combined with other health sector interventions and development such as hygiene, sanitation and education has led to a substantial reduction in mortality among under five children. However, disparities in vaccination coverage continue to exist across and within several nations [1]. The World Health Organization aims to make immunization services available to everyone, everywhere to save over 50 million lives by 2030 [2]. Immunization increases economic growth, productivity, and educational achievement and saves infant lives [3]. Low and middle-income countries generally have low immunization rates; in 2021 ten of these countries accounted for over 60% of the unvaccinated or under-vaccinated children. (Angola, Brazil, the Democratic Republic of Congo, Ethiopia, India, Indonesia, Myanmar, Nigeria, Pakistan, and the Philippines) [2].

A survey carried out in sub-Saharan countries between 2010 and 2016 found that, on average, 58.9% of children under five years had received all recommended vaccinations; this figure varies from 24.2% in Nigeria to 81.3% in Burkina Faso [4]. Another study showed that Africa has the highest rate of under and unvaccinated children worldwide. Approximately 12.7 million children were under-vaccinated in 2021, including 8.7 million “zero-dose” children or children who did not receive a single shot. Africa is home to half of the top 20 nations in the world with the highest percentage of zero-dose children. Among these nations, Nigeria and Ethiopia have the highest number of zero-dose children [5].

According to 2019 data, only 47% of Ethiopian children aged between 12 and 23 months received all recommended vaccines. Administrative regions vary in terms of the overall vaccination rate, for instance, in the Afar region, it is 21%, whereas in the Amhara regional state, it is 89% [6]. Ethiopia has been supplying 10 antigens (OPV, BCG, Pentavalent, PCV, Rota and Measles vaccine) since 2019 that address the main causes of childhood illness and death. The Ethiopian National Expanded Programme on Immunization (EPI) and the Global Vaccine Action set vaccine coverage goals of 90% nationally by 2020 [7].

The most common cause of severe pneumonia and pneumonia-related mortality globally is Pneumococcus bacteria or *Streptococcus pneumoniae*. The human nasopharynx is commonly asymptomatically colonized by pneumococci, especially in children [8]. Five percent of the 5.8 million children under five years who die each year are thought to be due to pneumococcal infections, according to estimates from the World Health Organization (WHO) [9]. Pneumonia is one of the main causes of mortality in Ethiopia for children under five, accounting for an estimated 40,000 deaths each year, and Ethiopia is ranked sixth among the top fifteen nations for pneumonia-related morbidity and death [10]. Vaccinating infants has been proven to be the most efficient approach to prevent diseases and lower the burden, mortality and consequences for both child and adult populations [11]. A study performed in Gambia showed that the introduction could prevent 117000 pneumococcal-related infections in under five children and save $4 million in health costs in health systems [12].

In Ethiopia, the percentage of children aged 12–23 months who completed their PCV regimens was only 49.1%. There were significant regional differences in PCV vaccination coverage, ranging from 17.5% in Afar to 91.4% in Addis Ababa [13]. The pneumococcal vaccine was introduced into the child immunization program in Ethiopia in 2011 with the aid of the GAVI vaccine alliance [13]. Starting in 2016, pneumococcal vaccine information was integrated and collected together with other vaccines in EDHS.

The main objective of the study was to assess the changes in the coverage of the pneumococcal vaccine and determine its associated factors. There is limited data on the changes in pneumococcal vaccine coverage and factors associated in Ethiopia. Therefore, this study assessed the changes in the coverage and potential factors linked with pneumococcal vaccine coverage, which would be essential to strengthen and revitalize existing healthcare policies and strategies to reduce the zero-dose or under immunized children across the country and identify areas of improvement to enhance the vaccine coverage.

## Materials and methods

### Study setting and design

Ethiopia is a country with the second-highest population in Africa, where achieving vaccination coverage plans remains challenging. This study was conducted based on secondary data obtained from the EDHS which covers all regional states and two city administrations. The EDHS is a comprehensive and nationally representative population and health survey conducted using a cross-sectional study design. The study participants were children aged between 12 and 23 months selected from two representative EDHS surveys in Ethiopia.

### Data

To analyze the change over time, data from two nationally representative surveys, the Ethiopian Demographic and Health Surveys (EDHS) in 2016 and 2019, were used. A total of 1929 and 1008 children aged 12–23 months were included in the 2016 and 2019 Demographic and Health Surveys, respectively.

### Sampling design

The Ethiopian Demographic and Health Survey is part of the worldwide MEASURE DHS project [14]. The sample at each survey was selected using a stratified and two-stage cluster design. Sampling weights are the adjustment factors applied to each case to adjust for differences in the selection probability. Thus, women (or children) sampling weights were used in all analyses to make sample data representative of the entire population. Apart from representativeness, the use of weighted estimation during data analysis provides some protection against potential biases introduced by informative sampling.

### Variables of the study

The outcome variable of the study was PCV vaccination status among children aged 12–23 months. Depending on the number of PCV vaccine doses the child received, the outcome variable vaccination status was defined as


$$\{\begin{array}{l} \begin{array}{cc} 0 & if\;no\;PCV\;vaccination\;was\;given\;at\;all \end{array}\\ \begin{array}{cc} 1 & if\;only\;the\;first\;dose\;of\;PCV\;was\;give \end{array}\\ \begin{array}{cc} 2 & if\;only\;the\;first\;two\;doses\;of\;PCV\;was\;given \end{array}\\ \begin{array}{cc} 3 & if\;a\;full\;dose\;(3\;doses\;of\;PCV)\;was\;given \end{array} \end{array}$$

The potential determinants of PCV coverage were first selected at the individual, household, and community levels based on related studies and data availability. To analyze the association between each of the selected possible determinants and an outcome of interest, only the 2019 EDHS was used. The 2019 EDHS is a recent mini-survey conducted in Ethiopia. Nine explanatory variables were considered as potential determinants of PCV vaccinations. Thus, the data file extracted from the mini-2019 survey data contained the following variables, as stated in Table 1.

**Table 1:** Description of the variables considered in the study.

Variable Name	Description
Residence	The type of residence (Urban/Rural)
Education	Mothers’ education (No education/Primary/Secondary and above)
Maternal age	Mothers age in years (15-19/20-24/25-34/35-49)
Birth order	the order a child is born (first/second or above)
Delivery place	Place of delivery (home/health facility)
ANC visit	Number of ANC visits (0/1/2/3/4^+^)
Marital status	Marital Status (Married/Unmarried)
Media exposure	Exposure to media (Yes/No)
Wealth	Wealth status (Poor/Medium/Rich)
PCV	PCV vaccine coverage (dose 0, dose 1, dose 2, dose 3)

### Statistical analyses

We used both STATA version 15 for descriptive and regression analysis and R version 4.2 statistical software for data management and visualizations. In this study, complete case data analyses were used using both descriptive and inferential statistics. We restored the survey’s representativeness by weighting the data using sample weights, main sampling units, and strata before the statistical analysis. Therefore, the PCV coverage was assessed using all children aged 12–23 months by taking the sampling weight into account. The degree of change in quantity over time was then calculated by using percentage changes between the survey years. Percentage change is defined as the relative change between an old value and its new value, expressed as a percentage of the old value. Additionally, a 95% confidence interval was calculated to determine the significance of the percentage changes. The percentage changes in PCV coverage stratified by region and residence were also depicted through tables and map. We used R software to create the maps using the base map shape file from https://data.humdata.org/dataset/cod-ab-eth. We adopted a multilevel model for identifying the factors associated with the uptake of PCV using the recent 2019 EDHS [14]. To measure the association between each of the possible determinants and an outcome of interest, first unadjusted multilevel ordinal logistic regression was used [15]. The p values resulting from this regression were considered to identify the candidate determinants for the final model. An unadjusted logistic regression in this analysis was computed by considering the outcome of interest and one of the potential risk factors at a time. Then, any variable having a significant pairwise test at the 25% level of significance was selected as a possible predictor for the final statistical model.

To identify the significant factors associated with the uptake of PCV among the selected candidates, a multilevel ordinal logistic regression model was used.

Let y_ij_ be the outcome used to indicate the number of doses of PCV that the i^th^ child received in cluster j and X_ij_ be the associated vector of covariates (i = 1, · · ·n_j_; j = 1, · · ·, k). Then, the multilevel ordinal logistic regression model that has 2 levels can be formulated as:


$$logit(P(y_{ij}>s│X_{ij}))=\beta_{0s}+X_{ij}\beta +u_{j},\;with\;u_{j}\sim N(0,\sigma_{u}^{2})$$

Where $P(y_{ij}>s│X_{ij})$ is the cumulative probability of the response in categories or above; *s* = 0, 1,2,3 denotes the 4 response categories “not received any dose of PCV”, “only one dose of PCV”, “two doses of PCV” and “full doses of PCV”; n_j_ is the number of children in the j^th^ cluster/EA; k is the number of clusters; and u_j_ is the random intercept used to absorb the correlation between subjects within a cluster.

### Estimation

When estimating parameters of the multilevel models, sampling weights are incorporated into the likelihood, which leads to a pseudo-likelihood. We then maximize the weighted pseudo-likelihood with respect to the regression parameters for the estimation of these parameters and their standard errors. A detailed explanation of the parameter’s estimation is presented in [15]. Every test was run with a significance threshold of 5%.

### Operational definition

**The pneumococcal vaccine** is currently conjugated in Ethiopia according to the EPI schedule, and the vaccine is delivered at 6 weeks, 10 weeks and 14 weeks of birth [13].

**The drop-out rate** is the rate difference between the initial vaccines (PCV-I) and the final vaccines (PCV-III).

**Index child**: refers to a 12- to 23-month-old child randomly included in the study from a household. In cases where more than one eligible child was present in a given household, one of them was selected at random to be included in the study.

**Household wealth index**, a composite measure of the socio economic status of the household used for ranking the households from poorest to richest in five wealth quantiles. It is measured based on asset ownership and housing characteristics including a source of drinking water, type of toilet facilities, type of cooking fuel, materials used for housing construction, ownership of land, and other assets [13]. For statistical analysis purposes, we classified the wealth index into three groups: poorest and poor wealth indices grouped into poor; richest and rich wealth indices grouped into rich; and medium income.

## Results

### Description of the study participants

According to the 2016 EDHS, 16650 households with 10006 alive children were included in the study. Among this, 2037 children were aged between 12–23 months. Similarly, in 2019 EDHS, 8663 households and 5415 alive children were included. Of this,1068 children were between aged 12–23 months. A total of 1,929 children from the 2016 EDHS and 1,008 children from the 2019 EDHS aged 12–23 months were considered for the analysis. Most of the participants were rural residents, with 88.4% in the 2016 survey and 69.5% in the 2019 survey. Among the children aged 12–23 months in each survey, 62.7% of their mothers had never been in school in the 2016 survey, compared to 45.2% in the 2019 survey. The majority of participants’ mothers were married (93.8% in the 2016 survey and 94.4% in the 2019 survey). Among the children included in the analysis, approximately 45.0% in the 2016 survey were from households with poor economic status, while it was 42.0% in the 2019 survey. The proportion of mothers who received at least one antenatal care from a skilled provider increased over time, from 65.0% in 2016 to 76.0% in 2019. In each survey, approximately half of the children’s mothers were aged between 25 and 34 years. The majority of the participants’ mothers and their family members did not have media exposure in either survey year. The number of home deliveries decreased from 63.4% in 2016 to 45.2% in 2019. The proportion of children born first was 18.6% in the 2016 survey and 23.5% in the 2019 survey, as stated in Table 2.

**Table 2:** Sociodemographic characteristics of the study participants.

Characteristics	Category	2016		2019	
		Number of Children (unweighted)	Percentage (weighted)	Number of Children (unweighted)	Percentage (weighted)
Residence	Urban	409	11.6%	267	30.5%
	Rural	1,520	88.4%	741	69.5%
Education	No education	1,170	62.7%	493	45.2%
	Primary	508	28.8%	342	40.6%
	Secondary & higher	251	8.5%	173	14.2%
Religion	Orthodox	607	34.8%	324	37.3%
	Catholic	15	1.4%	8	0.5%
	Protestant	346	22.2%	177	25.8%
	Muslim	921	39.2%	481	34.1%
	Traditional	26	1.8%	13	2.1%
	Other	14	0.6%	5	0.3%
Maternal Age	15–19	97	4.2%	66	7.1%
	20–24	449	20.7%	232	23.0%
	25–34	978	52.5%	532	50.5%
	35–49	405	22.6%	178	19.5%
Birth order	First	416	18.6%	229	23.5%
	Second and above	1,513	81.4%	779	76.5%
Place of Delivery	Home	1,101	63.4%	431	45.2%
	Health Facility	828	36.6%	577	54.8%
ANC visit	0	592	35.5%	245	24.1%
	1	91	3.8%	34	3.7%
	2	140	6.7%	80	6.1%
	3	310	17.6%	199	20.0%
	4+	796	36.6%	450	46.1%
Marital Status	Married	1,801	93.8%	940	94.4%
	Unmarried	128	6.2%	68	5.6%
Media exposure	Yes	631	30.4%	402	39.1%
	No	1,265	69.6%	598	60.9%
Wealth	Poor	979	44.9%	469	41.9%
	Medium	279	22.5%	136	17.4%
	Rich	671	32.6%	403	40.7%

### Pneumococcal vaccine coverage

Among children aged 12–23 months, 33% did not receive any dose of PCV in 2016, while 6.5% received only one dose of PCV, 11.4% received only two doses of PCV, and 49.1% received a complete series (i.e., three doses) of PCV. In contrast, in the 2019 survey, 26.4% of children in the same age group did not receive any dose of PCV, 5.2% received only one dose of PCV, 8.5% received only two doses of PCV, and the remaining 59.8% received complete (or three doses) PCV. Thus, the dropout rate decreased from 18% in 2016 to 14% in 2019.

The overall coverage for each PCV dose in both survey years is presented in Table 3. Generally, we observed significant steady progress in the progression of each dose of PCV coverage over time. The percentage of children aged 12-23 months who received the first dose of PCV increased from 67% in 2016 to 74% in 2019, reflecting a statistically significant 9.8% increase [95% CI: 2.1–18.1]. Similarly, the percentage of children aged 12–23 months who received the first 2 doses of PCV increased from 61% in 2016 to 68% in 2019, a significant 12.9% increase [95% CI: 3.9–22.9]. The percentage of children aged 12–23 months who received complete doses of PCV vaccinations increased from 49.1% in 2016 to 59.8% in 2019, a significant 21.8% increase [95% CI: 9.8-35.2]. Table 3 also presents the coverage of each dose of PCV vaccination stratified by the children’s place of residence across the two survey years. The highest percentage increase with respect to the coverage of the first and second doses of PCV vaccination across the two survey years was observed in urban residents compared to rural residents. However, the percentage increase in the uptake of the third PCV dose (complete doses) was higher in rural residents than in urban residents.

**Table 3:** Coverage of each dose of PCV vaccination in both survey years.

Doses	Residence	Year		Overall Percent Change (95% CI)
		2016	2019	
PCV-1	Urban	81.4 [67.2, 90.3]	87.4 [78.7, 92.9]	9.8 [2.1, 18.1]*
	Rural	65.1 [60.6, 69.4]	67.5 [61.0, 73.4]	
	Overall	67.0 [63.7, 70.1]	73.6 [69.3, 77.5]	
PCV-2	Urban	78.6 [69.6, 85.4]	85.2 [76.5, 91.0]	12.9 [3.9, 22.9]*
	Rural	58.1 [54.5, 61.6]	60.9 [55.8, 65.9]	
	Overall	60.5 [57.1, 63.7]	68.4 [63.8, 72.6]	
PCV-3	Urban	72.9 [63.6, 80.5]	77.9 [67.6, 85.6]	21.8 [9.8, 35.2]*
	Rural	45.9 [42.5, 49.6]	51.9 [46.7, 57.1]	
	Overall	49.1 [45.8, 52.5]	59.8 [55.0, 64.5]	

*: p-value < 0.05, PCV-1: pneumococcal conjugate vaccine dose 1, PCV2: pneumococcal conjugate vaccine dose 2, PCV-3: pneumococcal conjugate vaccine dose 3.

In both survey years, children in urban areas were more likely to receive all PCV doses (PCV-1-PCV-3) of vaccination than children in rural areas. The proportion of children who received a third (complete) dose of PCV was 73% in urban areas and 46% in rural areas. The dropout rates of PCV vaccinations from the first to second doses and second to third doses were much higher in rural areas than in urban residents in both survey years, as shown in Table 3.

**Fig 1:**
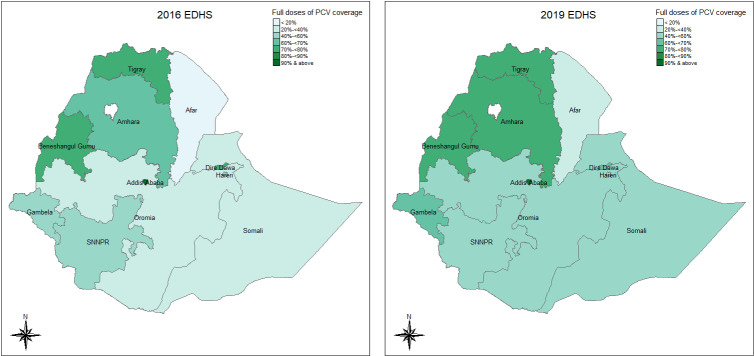
Regional distribution of full-dose PCV coverage in both survey years. (Source of the base map shape file: https://data.humdata.org/dataset/cod-ab-eth).

The distribution coverage of the three complete doses of PCV vaccinations across regions for both survey years is presented and compared (2019 vs 2016) in Fig 1. In the 2016 survey, the highest complete three doses of PCV vaccine coverage was observed in the Addis Ababa (91%), Tigray (78%), Dire Dawa (75%) and Benishangul Gumz (71%) regions, whereas the lowest coverage was observed in the Afar (18%), Somali (35%) and Oromia (38%) regions. According to the 2019 survey, the highest complete PCV vaccine coverage was observed in the Addis Ababa (93%), Tigray (78%), Amhara (78%) and Benishangul Gumz (75%) regions. All regions except Somali, Harari, and Dire Dawa showed improvement in completed (three dose) PCV vaccine coverage. However, a statistically significant percent increase in complete PCV vaccine coverage was observed in the Amhara (28.7% with 95% CI [9.6, 51.2]) and Oromia (37.3% with 95% CI [9.2%, 72.7%]) regions. On the other hand, a decrease (but not statistically significant) in complete PCV vaccine coverage was observed in Somali (from 34.9% in 2016 to 22.9% in 2019), Harari (from 58.6% in 2016 to 53.8% in 2019) and Dire Dawa (from 75.3% in 2016 to 68.2% in 2019).

### Determinants of pneumococcal vaccine coverage in Ethiopia

Based on the unadjusted multilevel ordinal logistic regression models and using a p-value cutoff of 0.25, all factors except marital status and media exposure were considered potential determinants of uptake of more doses of PCV vaccination. The results of the multilevel ordinal logistic regression model, after correcting for potential explanatory variables (adjusted) and when taking just one predictor variable at a time (unadjusted), are presented in Table 4.

**Table 4:** Estimates of the parameters from the multilevel ordinal logistic regression model of PCV vaccine recipients in Ethiopia using the 2019 EDHS.

Factors	Categories	COR [95% CI]	AOR [95% CI]
Place of Residence	Urban	ref	ref
	Rural	0.138 [0.064, 0.297]	0.307 [0.127, 0.742]*
Maternal education	No education	ref	ref
	Primary	1.673 [0.996, 2.811]	1.147 [0.623, 2.111]
	Secondary and above	2.313 [1.116, 4.794]	1.094 [0.486, 2.458]
Place of delivery	Home	ref	ref
	Health Facility	4.163[2.178, 7.958]	2.350 [1.132, 4.882]*
ANC Visits	No ANC	ref	ref
	1	0.467 [0.054, 4.035]	0.359 [0.050, 2.564]
	2	6.083 [2.046, 18.086]	4.213 [1.410, 12.584]*
	3	4.879 [1.711, 13.912]	2.931 [1.059, 8.109]*
	4 and above	6.687 [3.064, 14.593]	3.612 [1.639, 7.960]*
Birth order	First order	ref	ref
	Second and above	1.515 [0.759, 3.023]	2.519 [1.143, 5.548]*
Wealth	Poor	ref	ref
	Middle	1.014 [0.462, 2.226]	0.798 [0.352, 1.806]
	Rich	2.887 [1.449, 5.754]	1.361 [0.589, 3.143]
Region	Tigray	ref	ref
	Afar	0.039 [0.012, 0.127]	0.040 [0.011, 0.151]*
	Amhara	0.763 [0.224, 2.597]	1.021 [0.298, 3.496]
	Oromia	0.284 [0.089, 0.907]	0.354 [0.102, 1.229]
	Somali	0.038 [0.011, 0.136]	0.084 [0.022, 0.331]*
	Benishangul Gumz	0.817 [0.229, 2.920]	0.597 [0.152, 2.339]
	SNNPR	0.186 [0.062, 0.559]	0.218 [0.069, 0.683]*
	Gambela	0.313 [0.096, 1.022]	0.169 [0.046, 0.613]*
	Harari	0.191[0.054, 0.680]	0.101[0.026, 0.393]*
	Addis Adaba	6.424 [1.503, 27.453]	1.179 [0.222, 6.278]
	Dire Dawa	0.616 [0.217, 1.746]	0.322 [0.099, 1.052]
Random effect	Var (EA)		2.068 [1.264, 3.385]*

*: p-value < 0.05 SNPPR: south nation nationality and people regions, EA: enumeration area, COR: crude odd ratio, AOR: adjusted odds ratio, ref: reference.

Subject to the enumeration area (EA) level random effects, there were substantial differences in the likelihood of receiving more doses of PCV vaccines according to place of residence, birth order, place of delivery, ANC visits, and region.

Children in rural areas are 69% less likely to receive more doses of PCV vaccinations than those who live in urban areas (AOR = 0.307, 95% CI: 0.127–0.742). Second- or higher-order births are associated with a higher uptake of doses of PCV vaccinations (AOR = 2.519, 95% CI: 1.143–5.548]). Compared to children born at home, children born in health facilities were 2.35 times more likely to receive greater doses of the PCV vaccination (AOR = 2.350, 95% CI: 1.132–4.882). Children born to women who had more ANC visits were significantly more likely to receive complete PCV coverage. The likelihood of receiving high doses of PCV vaccinations was lower in the Afar, Somali, SNPPR, Gambela, and Harari regions than in the Tigray region, which showed the greatest uptake of the vaccine. However, maternal education and wealth were not significantly associated with PCV vaccination uptake (Table 4).

## Discussion

The study found a significant change in pneumococcal vaccination coverage from the 2016 EDHS to the 2019 EDHS. Place of residence, antenatal care, birth order, and place of delivery were found to be significantly associated with pneumococcal vaccine uptake in Ethiopia. In this study, we investigated the geographical distribution, epidemiological changes, and factors that determine the uptake of pneumococcal vaccines in Ethiopia.

The prevalence of completed (3-dose) vaccine coverage in 2016 was 49.11% and 59.8% in 2019, with a 21.8% change. This result is consistent with a study performed on the uptake of new vaccine reports in Ethiopia,[13]. In another study performed in Cameroon, the national pneumococcal vaccine coverage was 84% in 2017 and 79% in 2018 [16]. Few studies at the subnational level in Ethiopia have shown that pneumococcal vaccine coverage is 88% in Demba Gofa District, Southern Ethiopia and 76.7% in northern Gonder Ethiopia [17,18]. This pneumococcal vaccination coverage difference can be explained by geographical variation, different levels of health literacy and socioeconomic status across the country. Despite this variation, the national pneumococcal vaccine coverage increased nationwide from 49.11% in 2016 to 59.83% in 2019 according to the EDHS survey.

The study found that the drop-out rate of the pneumococcal vaccine decreased from 18% in 2016 to 14% in 2019. A similar study performed at zonal hospitals in northern Ethiopia showed that the dropout rate of pneumococcal vaccine was 4.5% from PCV-1 to PCV-3 [18]. Another study done in southern Ethiopia showed that the dropout rate of pneumococcal vaccine was 7.4% from PCV-1 to PCV-3 [19]. Those studies demonstrated that a contributing reason to the incompleteness of immunization is the scarcity and lack of several vaccinations, such as PCV, pentavalent and polio relative to the number of eligible children. Despite these obstacles, the country’s pneumococcal vaccine drop-out rate is declining. This may be attributed to rising vaccination rates nationwide, increased access to healthcare, greater maternal education and media exposure.

The study discovered that the children of uneducated mothers had the lowest immunization probability compared with the children of educated mothers, even though this result was not statistically significant. This result is consistent with previous studies [19–24]. This may be the case educated women understand the importance of protecting the health of their children and more informed about immunizations. Promoting and supporting women’s education is fundamental. Similarly, the study found that increasing birth order is significantly associated with vaccine uptake and completeness. In contrast, the studies conducted in Kenya and Cameroon showed that birth order negatively affected child immunization [25,26].

In this study, place of residency was significantly associated with completeness of the pneumococcal vaccine. Living in rural areas has a 69% lower likelihood of completing the vaccine compared to urban residents [AOR = 0.307 (CI = 95% 0.127, 0.742)]. A similar study performed on vaccine coverage showed that urban residency has positive implications on vaccine utilization and completeness [26–28]. Different constraints may prevent rural women from using maternity and child healthcare services. One important obstacle may be the difficulty in vaccinating children due to inadequate health service accessibility [35]. According to the current study, increasing the number of antenatal care follow-up and health institution deliveries were significantly associated with the completion of the pneumococcal vaccine. This result is consistent with those of previously reported studies [29–34]. This might be due to health practitioners encourage women who attended ANC follow-up to protect their children and prenatal care counseling can help women remember to vaccinate their children.

The study discovered a strong correlation between geographical variance and PCV vaccine completeness. The areas of Addis Ababa, Amhara, Tigray, and Benishangul Gumz have high vaccination completion rates. Conversely, the regions of Afar and Somalia have the lowest vaccination rates. This result is in line with previous studies reports [20,30,35]. This could be owing to geographical and logistics challenges, as these regions are known for their limited healthcare infrastructures, making it difficult to reach remote areas with vaccination programs, which could impede vaccine delivery. Additionally, a shortage of health professionals may make it difficult to obtain the latest data on vaccines and their benefits, which could result in people not following advised vaccination schedules [36]. This study has limitations due to the cross-sectional structure of the survey; we are unable to demonstrate the temporal relationship between independent variables and pneumococcal vaccine coverage among children aged 12–23 months. There may be a possibility of recall bias because the EDHS survey was based on the reports of the respondents.

## Conclusion

The coverage of the pneumococcal vaccine significantly changed over time across the country. Place of residency, antenatal care, birth order and place of delivery and regional variations were significantly associated with vaccine completeness. Despite these changes, Ethiopia is still far from achieving national immunization goals which target national vaccination coverage including pneumococcal vaccine of 90% by 2020. Reducing the observed regional inequalities and addressing the factors associated with coverage in pneumococcal vaccination could reduce morbidity and death due to vaccine-preventable illnesses.

## Abbreviation:

ANC: Antenatal care
EA: Enumeration area
EDHS: Ethiopia Demographic and Health Survey
EPI: Expanded Program on immunization
PCV: pneumococcal conjugate vaccine
SEA: Southeast Asia
SSA: Sub-Saharan Africa
WHO: World Health Organization
